# Tetrahydrocurcumin Lipid Nanoparticle Based Gel Promotes Penetration into Deeper Skin Layers and Alleviates Atopic Dermatitis in 2,4-Dinitrochlorobenzene (DNCB) Mouse Model

**DOI:** 10.3390/nano12040636

**Published:** 2022-02-14

**Authors:** Komal Saini, Nancy Modgill, Kamalinder K. Singh, Vandita Kakkar

**Affiliations:** 1 Department of Pharmaceutics, University Institute of Pharmaceutical Sciences, Panjab University, Chandigarh 160014, India; komalsainiks@gmail.com (K.S.); nancymodgill92@gmail.com (N.M.); 2 School of Pharmacy and Biomedical Sciences, Faculty of Clinical and Biomedical Sciences, University of Central Lancashire, Preston PR1 2HE, UK; 3 UCLan Research Centre for Smart Materials, University of Central Lancashire, Preston PR1 2HE, UK; 4 UCLan Research Centre for Translational Biosciences and Behavior, University of Central Lancashire, Preston PR1 2HE, UK

**Keywords:** tetrahydrocurcumin, lipid nanoparticles, atopic dermatitis, skin targeting, dermatokinetics, skin hydration

## Abstract

Treatment of atopic dermatitis (AD) is challenging due to its complex pathophysiology. Tetrahydrocurcumin (THC) a polyphenolic, colorless compound that is more polar than curcumin. It possesses superior anti-inflammatory properties and has a clinical advantage over curcumin. The present study investigated the therapeutic effectiveness of THC solid lipid nanoparticle (THC-SLN)-based gels in AD. THC-SLNs prepared using microemulsification resulted in a particle size of 109.2 nm as determined by nanoparticle tracking, and FTIR confirmed the entrapment of drug within the lipid matrix. THC-SLNs greatly enhanced skin hydration when tested both ex vivo and in vivo in Lacca mice. Deeper skin penetration was clearly established using dermatokinetics and CLSM. The in vivo pharmacodynamics of THC-SLNs gel in 2,4-dinitrochlorobenzene (DNCB)-induced AD mice showed enhanced bioactivity; reduced levels of TNF-α and IL-6; and complete healing, as evident from histopathological studies. Thus, the novel topical THC-SLN gel has potential to emerge as a safe alternative to conventional corticosteroids for AD and other skin disorders with overbearing inflammation.

## 1. Introduction

Atopic dermatitis (AD) is a common, intensely pruritic skin disorder that is challenging and frustrating to treat. It is a chronic inflammatory skin condition with a wide variety of clinical manifestations. The mechanism underlying the pathogenesis of AD remains unclear, but numerous studies have demonstrated the integral involvement of immunopathology, genetic disposition, emotional and environmental stimuli in the development of AD and its progression. Ad is also associated with the reduced water content of the stratum corneum that results in dry skin and relapsing eczema. Further, the roles of reactive oxygen species (ROS) have been studied in AD and other skin diseases to some extent, but its importance in AD has rarely been investigated [1].

Topical corticosteroids are the first-line therapies for AD but may lead to thinning of the skin on chronic usage [2]. Calcineurin inhibitors such as tacrolimus and pimecrolimus have attracted a lot of attention over recent years for efficacy in treating AD, but are not recommended for long term or chronic therapy due to the high associated risks of skin cancer and lymphoma [3]. Lack of safe available therapies to cater to a complete cure of AD have led researchers to explore alternative therapeutics from natural sources that are expected to not be allergenic or harmful, or not accumulate toxic metabolites [4].

Curcumin, a naturally occurring anti-inflammatory agent, has been used for treating various medical conditions for several years [5]. Several experimental and pharmacological trials have demonstrated its efficacy as an anti-inflammatory agent [6]. However, its use for inflammatory conditions of the skin may be restricted due to the strong yellow color of the pigment which results in staining of the skin and clothes post topical application.

Tetrahydrocurcumin (THC), a polyphenolic, colorless and more polar compound than curcumin, is a hydrogenated metabolite of curcumin. THC demonstrates similar physiological and pharmacological properties to those of native curcuminoids [7,8]. THC possesses superior pharmacological efficacy and clinical advantages over curcumin, such as better antitumor activity; antioxidant, anti-diabetic, and anti-aging effects; and wound healing properties [8]. Topical application of a THC calcium mineral complex showed decreased expression levels of inflammatory genes, such as cyclooxygenase-2 (COX-2) and interleukin-1-α (IL-1α) in keratinocytes; however, there are no investigations reported on use of THC for AD.

Inflammation is a defense mechanism against external changes or cellular injury that induces the release of mediators of the immune system at the site of inflammation [9]. Inflammatory skin disorders such as AD, psoriasis and rosacea are the common disease that led to inflammation that damages normal tissue. Inflammation plays an important role in defense against injury to cells: external changes and mast cells release the mediators that cause chronic inflammation [10]. The pro-inflammatory cytokines, for example, IL-1α, IL-1β, IL-6,8 and tumor necrosis factor-α (TNF-α), are secreted by immune cells and promote inflammation [11]. Thus, therapeutic molecule that can reduce the excess release of proinflammatory cytokines can interrupt the inflammatory process and could offer an effective healing strategy for reduction of skin inflammation [12].

During the past few decades, lipid-based nanosized particles have gained momentum due to their multiple advantages, presented as promising cutting-edge technology for treatment of skin diseases [13]. Lipid nanoparticles (LNs) enable one to target the drug payload to deeper skin layers, overcoming skin barriers and enhancing drug delivery to the skin [14]. They provide localized access to pathological sites through enhanced drug permeation into the skin in a controlled manner, and have shown significant therapeutic effectiveness in AD [15]. The highly specific surface area of LNs also facilitates enhanced bioavailability in the skin and offers protection for drug molecules from degradation [16].

Our previous study reported the development of THC-loaded solid lipid nanoparticles (THC-SLNs) and revealed their superior ability to penetrate the skin ex vivo, along with their in vivo efficacy, in a wound healing model [17]. The studies on the excision wound mouse model emphasized that THC-SLN gel not only has significant antioxidant and anti-inflammatory properties, but also possesses remarkable wound healing capabilities. The wound healing proficiency of THC could be attributed to its enhancing of epithelialization, cellular proliferation, the formation of granulation tissue and synthesis of collagen tissue, after dermal application, owing to its angiogenic potential.

With THC being a potent antioxidant with superior anti-inflammatory power and AD being essentially an inflammatory disease, the purpose of the present study was to investigate the therapeutic effectiveness of THC-SLN gel against AD. THC-SLNs were prepared with a modified microemulsification method, followed by a high-speed homogenization (HSH) technique, which resulted in high drug loading. We report (a) confirmation of the entrapment of drug into the SLNs using FTIR, and the particle size distribution and concentration of SLNs in the dispersion using nanoparticle tracking analysis; (b) ex vivo and in vivo evaluations of skin hydration using THC-SLN gel vs. a marketed ointment; (c) a proof-of-concept study of deeper skin permeation and ex vivo targeting using dermatokinetic modelling and cutaneous uptake of SLN in vivo in Lacca mice using confocal laser scanning microscopy (CLSM); and (d) an in vivo pharmacodynamic assessment (including biochemical, molecule markers and histopathological) using a murine disease model of AD induced by 2,4-dinitrochlorobenzene (DNCB) to establish the enhanced therapeutic efficacy of THC-SLN gel. All animal studies were approved by the Institutional Animal Ethics Committee (IAEC) (PU/IAEC/S/14/59) of Panjab University, Chandigarh.

To best of our knowledge, this is the first report describing the therapeutic effectiveness of THC and its nanoparticles for treatment of AD. The results of our research provide valuable evidence, which supports THC-SLN gel as a potential candidate for improved treatment of AD.

## 2. Materials and Methods

### 2.1. Materials

Tetrahydrocurcumin was kindly provided as a gift sample by Sanat Pharmaceuticals Ltd. (New Delhi, India). Carbopol^®^ 934, disodium hydrogen phosphate, sodium dihydrogen phosphate and Tween 80 were purchased from Central Drug House (P) Ltd. (New Delhi, India). Compritol^®^ 888ATO and Phospholipon 90 G were received as a gift sample from Panacea Biotec (Lalru, Punjab, India) and Gattefosse SAS, France, respectively. Chloroform and diethyl ether were purchased from Sisco Research Laboratories (Mumbai, India). Ethylene diamine tetra acetic acid and triethanolamine were provided by SD Fine Chemicals (Mumbai, India). Formalin and hydrochloric acid were supplied by Qualigens Fine Chemicals (Mumbai, India). Ellman reagent and 2,4-dinitro-chlorobenzene were obtained from Himedia Laboratories Ltd. (Mumbai, India). Methanol and polyethylene glycol were obtained from Fisher Scientific (Mumbai, India). 5,6-Carboxyfluoroscein was purchased from Sigma-Aldrich, Co., Burlington, MA, USA. The RayBio^®^ Mouse IL-6 ELISA Kit was provided by Ray Biotech, Inc., Norcross, GA, USA.

### 2.2. Production of Tetrahydrocurcumin-Loaded Solid Lipid Nanoparticles (THC-SLNs)

(a) Preparation of the THC-SLN dispersion was carried out according to previously reported method [17,18,19,20] with several modifications. In brief, accurately weighed amounts of Tween 80 (30%) and phospholipon 90 G (2%) were simultaneously dispersed in water and heated to the melting temperature of the lipid. Compritol^®^ 888 ATO (7.27%) was separately melted at 82–85 °C. THC (0.4%) was dispersed in the aqueous system, mixed and then added directly into melted lipid to obtain a clear microemulsion while continuously stirring on a magnetic stirrer. The hot microemulsion was then squirted all at once into an equivalent quantity of chilled water (2–5 °C) under continuous homogenization (13,000 rpm) for 20 min using a high-speed homogenizer (T18 Digital ULTRA-TURRAX^®^ IKA, Werke Staufen, Germany), resulting in the formation of SLNs. The resultant THC-SLNs were stored at room temperature.

(b) For preparation of fluorescent dye-loaded SLN, 5,6-carboxyfluorescein (CFC) was used in place of THC. All other steps of the preparation remained the same. Then, dye-loaded nanoparticles were characterized regarding particle size and drug content.

### 2.3. Preparation of THC-SLNs-Based Gel

To facilitate better spreadability on topical application and enhance patient compliance [21], a THC-SLN dispersion was loaded in a Carbopol (2% *w*/*v*) hydrogel. Briefly, the polymer was added to water (25 mL) under stirring and kept for hydration for 12 h. Triethanolamine (0.75 g), was used to neutralize the Carbopol under continual stirring to yield a uniform translucent gel. THC-SLNs (25 mL) were poured into this Carbopol gel and blended gently to achieve a homogenous white gel consisting of 1% *w/v* of Carbopol and 0.2% *w*/*w* THC. Gel containing free drug was prepared by adding THC at same concentration as contained in THC-SLNs to the Carbopol (1% *w*/*v*) gel and mixed to ensure homogeneity [17].

### 2.4. Characterization of THC-SLNs

#### 2.4.1. Total Drug Content (TDC) and Entrapment Efficiency (EE)

TDC and EE were determined by the procedure previously reported [17] and calculated using Equations (1) and (2), respectively.

(1) $$\%\;\mathrm{EE}=\left\{\frac{\left(\mathrm{TD}-\mathrm{FD}\right)}{\mathrm{TD}}\right\}\times 100$$

(2) $$\%\;\mathrm{TDC}=\left\{\frac{\left(\mathrm{THC}\;\mathrm{concentration}\;\mathrm{after}\;\mathrm{processing}\right)}{\left(\mathrm{THC}\;\mathrm{added}\;\mathrm{concentration}\;\mathrm{before}\;\mathrm{processing}\right)}\right\}\times 100$$

#### 2.4.2. Nanoparticle Tracking Analysis

The particle sizes of THC-SLNs and blank SLNs were measured using a Nanoparticle Tracking and Analysis (NTA) system (NanoSight 300 Malvern, Groovewood Road, Malvern, UK) equipped with a scientific CMOS camera, a blue laser module 488 and NTA software 3.3. The sample was diluted in double distilled water and injected by using the disposable syringe into the NS 300 sample laser module (sample holder) with a syringe pump speed set at 70. The detection threshold of the NTA software was set to 4, and the maximum jump distance and the minimum track segment length were both set to auto. The data were generated and expressed as mean (average particle size measurement) or mode (most frequently observation). The former was recorded for each sample, and the modal value (most frequent particle size) was taken as the nanoparticle size. In NTA, the Stokes–Einstein equation is used to calculate the particle size [22].

#### 2.4.3. Fourier Transform Infra-Red Spectroscopy (FTIR)

FTIR is a method conducive for the analysis of a bulk material and its constituent nanoparticles to confirm the entrapment of drug within the lipid matrix [23]. FTIR analysis was performed for the free drug, lipid, physical mixture of drug and lipid and THC-SLN dispersion on a Perkin–Elmer Spectrum RX-IFTIR (Waltham, MA, USA). Samples were measured for percentages of transmittance ranging between 400 and 4000 cm^−1^.

### 2.5. Skin Hydration Studies

#### 2.5.1. Ex Vivo Skin Hydration Studies

Occlusion tests were performed in accordance with the reported method [17,24]. Purified water (50 g) was placed in a beaker, covered with lacca mouse skin and sealed properly. THC-SLN gel, blank SLN gel, plain Carbopol gel base (without THC-SLNs) and reference Tacroz^®^, 250 mg in each case, were spread on the surface of the mouse skin uniformly and kept at 32 °C and 60 ± 5% RH. The beakers’ weights were recorded after 24 and 48 h. The percentage of water loss through the mouse skin was calculated, and occlusion factor F was determined as per the expression given in Equation (3).

F = (A − B)/A × 100(3)

where A and B represent the water loss without sample and with sample, respectively.

#### 2.5.2. In Vivo Skin Hydration Studies

The skin hydration effects of selected formulations were investigated in vivo in lacca mice (Institutional Animal Ethics Committee (IAEC) (PU/IAEC/S/14/59) of Panjab University, Chandigarh, India). The animals were housed under controlled laboratory conditions. THC-SLN gel, THC-SLN dispersion, free THC in Carbopol gel, blank SLN gel and reference Tacroz^®^ Forte ointment, 250 mg in each case, were applied to shaved skin of male lacca mice. The naive (control) skin served as a reference. After 24 h, the animals were sacrificed humanely, and the skin was isolated to remove the fatty layer with a surgical blade [25]. The skin hydration potential was evaluated in terms of the thickness of the skin, which was measured using vernier calipers [26].

### 2.6. Skin Penetration of THC-SLNs

#### 2.6.1. Ex Vivo Penetration and Dermatokinetics

Ex-vivo permeation studies were conducted on Franz diffusion cells using excised skin of Wistar rats as the membrane. The skin was treated with THC-SLNs and free THC in gel as per the procedure given in our previous work [17]. After the treatment, whole skin was removed from the Franz diffusion cells at the respective sampling times of 0.5,1, 1.5, 2, 4 or 6 h. The skin samples were cleaned and washed three times to remove formulation adhering to it. Then, the samples were left in warm water (about 60 °C) and the two layers, i.e., epidermis and dermis, were separated carefully. Both layers were sliced into small pieces, then vortexed with methanol (5 mL) for 24 h for drug extraction. The methanolic solution was filtered and filtrate was analyzed for THC using UV spectrophotometer at λ_max_ of 282 nm.

The drug concentration time profile was fitted into one-compartment open model as per Equation (4).

(4) $$C_{\mathrm{skin}}=\frac{K_{p}C_{\max\left(e^{-K_{p}t}-e^{-K_{e}t}\right)}}{\left(K_{p}-K_{e}\right)}$$

where C_skin_ is the drug concentration in the skin layers, i.e., epidermis and dermis, at time t. K_p_ and K_e_ are the dermal permeation constant and skin elimination constant. C_max_ is the maximum concentration attained in skin.

Win-Nonlin Version 5.0 software was used to calculate various dermatocokinetic parameters, such as K_p_, C_max_, K_e_, t_max_ and area under the curve (AUC0-6 h) using the Wagner–Nelson method [27].

#### 2.6.2. In Vivo Penetration Using Confocal Laser Scanning Microscopy (CLSM)

Cutaneous uptake of THC-SLN dispersion was examined in vivo in lacca mice using CLSM. The animals were housed under controlled laboratory conditions and were given free access to food and water. 5,6-Carboxyfluorescein (CFC) was used as the fluorescent probe (4 µg/mL) in all formulations. The study was done in three groups: Group 1: Free CFC in Carbopol gel. Group 2: Blank SLNs along with free CFC in gel base. Group 3: CFC-loaded SLN (CFC-SLN) gel. The formulations were applied to skins of the mice (*n* = 3) for 24 h, after which the formulation was removed carefully to avoid any contamination, and each application area was excised from the humanely sacrificed animals and collected. Thereafter, the skin was rinsed with normal saline and sectioned. The sections were fixed in paraffin, and vertical slices were prepared of 25 mm thickness using a microtome. The fluorescence distribution was immediately investigated using CLSM (M/s Nikon C2si, Tokyo, Japan). The fluorescence was recovered in the red band by exciting the specimens at 487 nm, and fluorescence pictures were taken with 20× magnification. Fluorescence yield was evaluated by image treatment software, the integration of pixel brightness values (arbitrary brightness values, ABU) giving the relative dye content within skin [28,29].

### 2.7. Pharmacodynamic Activity in a Murine Atopic Dermatitis (AD) Model

#### 2.7.1. Effectiveness against AD

Female *Lacca* mice of average weight, 20–40 g, were used in this study. The animals were housed under controlled laboratory conditions. To induce AD-like symptoms, 1-chloro-2,4-dinitrobenzene (DNCB) was used to sensitize the hair-free skin of female *Lacca* mice [30,31].

Animals were randomly divided into 7 groups having three animals each (Table 1). During the first week, topical application of 100 µL DNCB (1% *w/v* in a 3:1 of acetone: olive oil) was performed on all the animals except the negative control group. After 1 week, all DNCB-treated groups except the negative group were further sensitized topically with a 50 µL (0.5% *w/v* in a 3:1 of acetone: olive oil) solution of DNCB once every other day for an extra 5 days. Post setting-in of AD, i.e., from the 13th day till the 22nd day, the skin samples were treated with THC-SLN dispersion, THC-SLN gel, Tacroz^®^ Forte ointment (contains 0.1% *w*/*w* tacrolimus), blank SLN gel or free THC gel containing 0.2% THC once daily topically on the skin—groups I, II, III, IV and V, respectively. Clinically, the severity of AD symptoms (erythema, edema, oozing or crusting, excoriation, lichenification, dryness) was observed, and effectiveness against AD was assessed in terms of SCORAD (scoring atopic dermatitis) index [32,33,34], as given in the Supplementary data. Images of representative mice with clinical AD symptoms from all groups were captured on several days (days 1, 2, 3, 4, 5, 6, 8, 9, 10, 12, 13, 15, 18 and 22). Post-treatment, at the end of day 22, animals were humanely sacrificed, and the dorsal side skin from all treatment groups was removed rinsed with ice-cold phosphate buffer saline, pH 7.4 (PBS), and thin sections were investigated histopathologically. The remaining portions of the skin from the animals (*n* = 3 sites) were washed with PBS and homogenized using a tissue homogenizer at the speed of 5000 rpm for 10 min, in 10% PBS for biochemical estimation and 40% PBS for determination of molecular markers [17]. After homogenization, samples were centrifuged at 10,000 rpm at 4 °C for 10 min, and the clear supernatant was used for analysis.

#### 2.7.2. Biochemical Estimation

Protein content was estimated using the Biuret method with bovine serum albumin as the reference protein [17]. Lipid peroxidation (LPO), glutathione (GSH) level and catalase activity were estimated as per standard referred techniques [35,36].

#### 2.7.3. Evaluation of Molecular Markers (IL-6 and TNF-α) in Skin

Molecular markers, interleukins-6 (IL-6) and tumor necrosis factor-α (TNF-α) were estimated using an enzyme-linked immunosorbent assay (ELISA) kit (R&D system, quantakine USA [37]. The samples were tested thrice, and ELISA assays were performed as per guidelines of the manufacturer with detection at 450 nm. The concentrations are expressed as picograms per milliliter.

#### 2.7.4. Histopathological Studies

The thin sections were stained with hematoxylin and eosin (H&E), and microscopic examination was carried out under a high-powered light microscope. The photomicrographs were taken and observed for any histopathological changes.

### 2.8. Statistical Analysis

All experimental data are expressed as mean ± standard deviation (SD) and were analyzed using one-way ANOVA. *p*-values ≤ 0.05 were taken as statistically significant.

## 3. Results

### 3.1. Formulation of Lipidic Nanoparticles Loaded with Tetrahydrocurcumin (THC-SLNs)

THC-SLNs were produced by a modified method of micro-emulsification to reduce the time for preparation by using a high-speed homogenizer at 13,000 rpm for 20 min [19,20]. Homogenization resulted in droplet breakup and regulated the size of the microemulsion. Droplet breakup is mainly dependent upon the amount of shear force applied for deformation, but its maintenance and avoidance of coalescence are established by the ability of the surfactant to adsorb on the surface of newly formed droplets, which depends upon the concentration and surface activity of the selected surfactant [38].

The concentrations of surfactants, Tween 80 (7.5%) and phosphatidylcholine (0.5%), were optimized and well within the specification of the USFDA for inactive ingredients [39,40]. Compritol^®^ 888 ATO was selected as the lipid constituent of the SLNs, as it is well established for topical delivery [41,42,43].

### 3.2. Characterization of THC-SLNs

#### 3.2.1. Total Drug Content (TDC) and Entrapment Efficiency (EE)

The EE of the THC-SLNs was improved to 83.10% ± 2.29% (*n* = 6) when a high-speed homogenization process was used, as compared to 69.56% ± 1.35%, which was previously reported with a mechanical mixing process [17]. The higher EE indicates the suitability of the method used for the preparation of SLNs. The high-speed homogenization required a shorter mixing time, and efficient mixing leads to a more uniform surfactant distribution, resulting in higher EE. The TDC of THC-SLNs and THC-SLN gel prepared by high-speed homogenization were estimated to be 93.6% ± 2.48% and 95.50% ± 2.78% respectively. High values of TDC are indicative of the insignificant losses incurred during the process of preparation of THC-SLNs by the high-speed homogenization technique.

#### 3.2.2. Nanoparticle Tracking Analysis (NTA)

**Figure 1:**
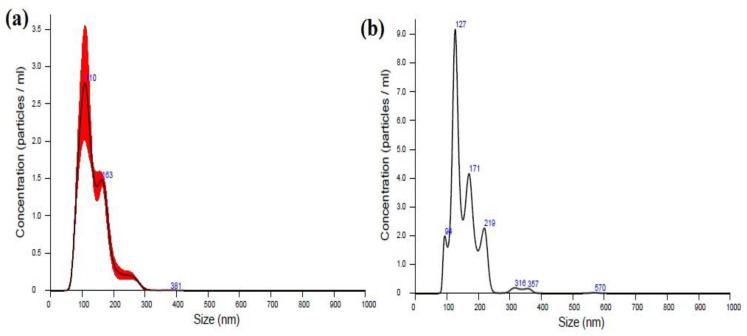
Nanoparticle tracking analysis of (**a**) THC-SLNs and (**b**) blank SLNs.

The size and concentration of SLNs was investigated with NTA. This method works on the principle of a laser illuminated microscopical system that allows one to image the Brownian motion of nanoparticles by the use of a charge-coupled device (CCD) camera. The nanoparticles were visualized and individually tracked by software with image analysis that analyzed the coefficient of diffusion for every particle and permitted the determination of its hydrodynamic radius [44]. The size distribution of nanoparticles with their corresponding video frames (Supplementary data video) and graphs (concentration vs. size) is shown in Figure 1. The particles were polydisperse: modal sizes were 109.4 and 126.5 nm for THC-SLNs and blank SLNs, respectively. This is in line with previous reports showing larger particles for blank nanostructured lipid carriers (NLCs) due to their lack of a dense drug core [45,46].

#### 3.2.3. Fourier Transform Infrared Spectroscopy (FTIR)

**Figure 2:**
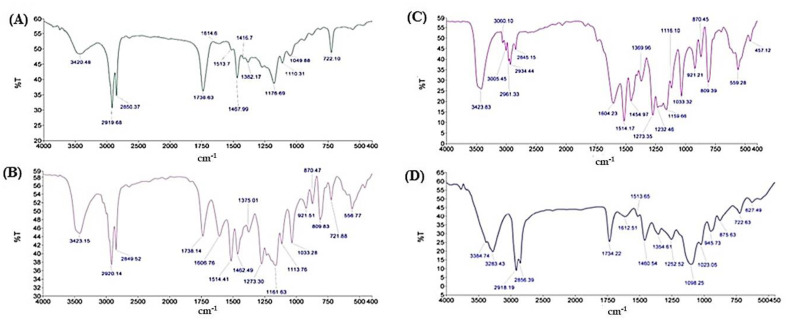
FTIR spectra of (**A**) pure THC (**B**) pure Compritol^®^ 888 ATO (**C**) physical mixture of THC and Compritol^®^ 888 ATO (**D**) THC-SLN dispersion.

FTIR was used to investigate the interplay and interactions between drug and lipid during the preparation of SLNs (Figure 2). The feature peaks, frequencies and areas give the differentiation of functional groups of lipid and THC. The characteristic peaks of THC (Figure 2A) appeared at 3427, 2933, 1733 and 1604 cm^−1^, which are associated with –OH stretching, aromatic –CH stretching, –C=O stretching and aromatic C=C stretching respectively. Compritol^®^ 888 ATO (Figure 2B) indicated the –OH stretching at 3420 cm^−1^ and aromatic –CH stretching at 2919 cm^−1^. The peaks of drug and lipid were retained in case of their physical mixture (Figure 2C) with a slight shift indicating no chemical interaction between them [47]. On the other hand, the characteristic peak THC was lacking and –OH stretching of Compritol^®^ 888 ATO shifted to 3384 cm^−1^ in case of THC-SLNs (Figure 2D). This confirmed the successful incorporation of THC into the lipid nanoparticles. These results are in consonance with a previous report [48].

### 3.3. Skin Hydration

#### 3.3.1. Ex Vivo Studies 

Upon dermal application, SLNs, with their tendency to form an adhesive layer on the skin, are capable of providing an occlusive effect. This leads to increased skin hydration. As a result, gaps between corneocytes widen and block the pores of skin layers, which ultimately assists with the penetration of a drug into deeper skin strata. Furthermore, the occlusive effect is strongly associated with the particle size of SLNs, as nanoparticles have been stated to be 15 times more occlusive than microparticles [49]. Additionally, particles <400 nm in size, including at least 35% lipids of high crystallinity, are reportedly more effective in terms of providing occlusive effects [49].

**Figure 3:**
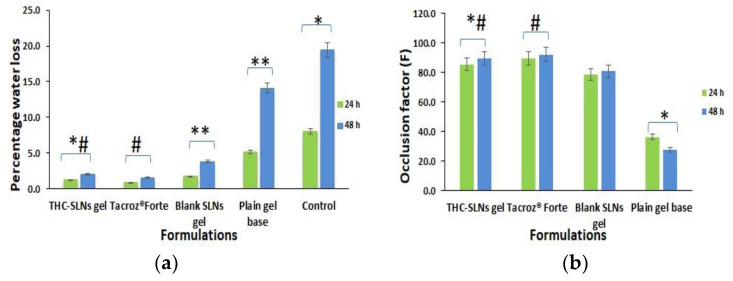
Graph depicting (**a**) percentage water loss with time and (**b**) occlusion factor, F, with time (each value represents mean ± SD (*n* = 3)). One-way ANOVA followed by Tukey’s test: * denotes a significant difference at *p* ≤ 0.05 from control group; ** denotes a significant difference at *p* ≤ 0.05 from plain Carbopol gel; # denotes no significant difference at *p* ≤ 0.05.

The ex vivo occlusion study revealed almost 4.29 and 6.98 times reductions in water loss for THC-SLN gel as compared to the plain Carbopol gel base after 24 and 48 h, respectively (Figure 3a). There was no statistically significant difference (*p* ≤ 0.05) between water loss via skin for THC-SLN gel and the reference ointment, Tacroz^®^ Forte. Blank SLNs also showed a decrease in water loss by 2.98 and 3.74 times in comparison to the plain Carbopol gel base after 24 and 48 h, respectively. Thus, THC-SLN gel could provide occlusivity equivalent to the ointment (Figure 3b) with a sustained effect, but without the greasiness of the ointment, as the gel is aqueous with a plain Carbopol gel base [50].

#### 3.3.2. In Vivo Studies

The skin hydration potential (SHP) was calculated in terms of thickness of skin measured with vernier calipers after treatment with THC-SLN gel, THC-SLN dispersion, free THC gel, plain gel or Tacroz^®^ Forte ointment, as compared to naive (control) skin as the reference (Table 2).

**Figure 4:**
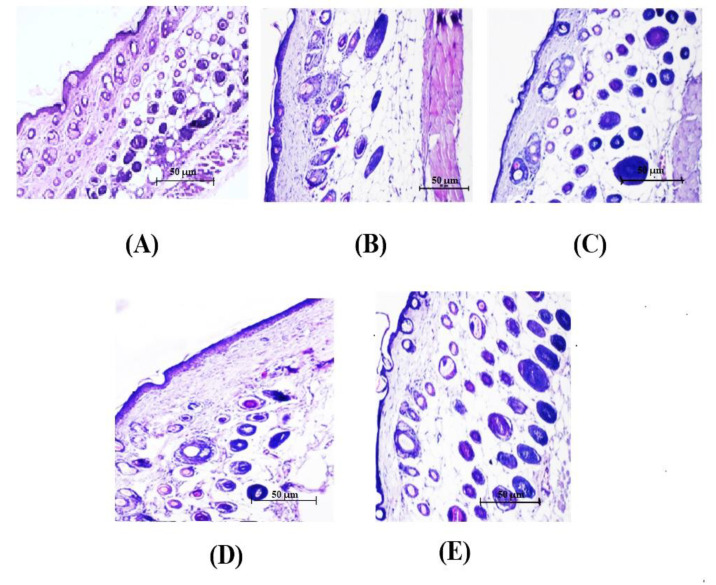
Photomicrographs of mouse skin illustrating the hydration of the skin. (**A**) Untreated skin; (**B**) skin treated with blank SLN gel base; (**C**) skin treated with THC-SLN dispersion; (**D**) skin treated with reference ointment, Tacroz^®^ Forte; and (**E**) and skin treated with THC-SLN gel.

The SHP of THC-SLN gel was found to be 2.16 times than of the control. THC-SLN dispersion and Tacroz^®^ Forte showed similar SHPs, about double the values for the control group. This was also evident from the photomicrographs showing visible differences in thickness of stratum corneum for the treated skin samples as compared to control (naïve) skin (Figure 4). The SHP of the THC-SLN gel was 1.44 times higher than that of the free THC gel (*p* ≤ 0.05), due to the occlusive nature of SLNs once incorporated into the gel [51,52].

### 3.4. Skin Penetration of THC-SLNs

#### 3.4.1. Ex Vivo Penetration and Dermatokinetics

**Figure 5:**
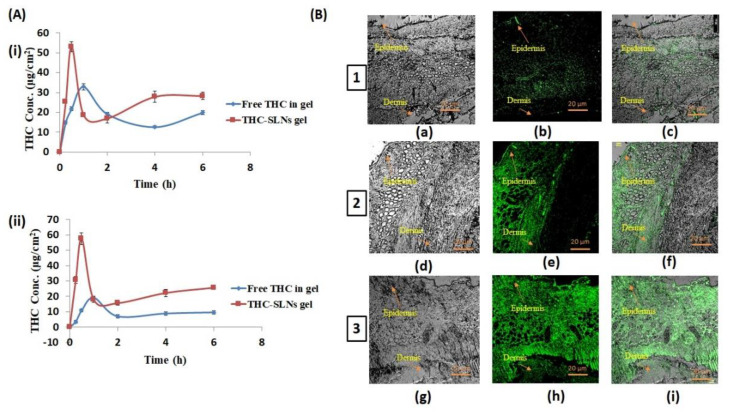
(**A**) (i) The THC concentration (µg/cm^2^) versus time (h) profile at various time points in the epidermal layer of the skin. (ii) The THC concentration (µg/cm^2^) versus time (h) profile at various time points in the dermal layer of the skin after treatment with THC-SLN gel and free THC in gel. All values are expressed as mean ± SD (*n* = 3). (**B**) Confocal laser scanning microscopy images showing the distribution of 5,6-carboxyfluorescein in the skin at the end of 24 h of treatment: (1) skin treated with free 5,6-carboxyfluorescein in a Carbopol gel base, (2) skin treated with free 5,6-carboxyfluorescein and blank SLN gel in combination and (3) skin treated with 5,6-carboxyfluorescein-loaded CFC-SLN gel. (Note: Images (a,d,g) were taken by bright-field microscopy; (b,e,h) indicate detection of 5,6-carboxyfluorescein; and (c,f,i) indicate the overlap of these two images).

Figure 5A(i,ii) depict the comparative drug concentration time profiles after treatment with THC-SLN gel and free THC gel in each of the layers of skin—i.e., the epidermises and dermises of Wistar rats, respectively. The THC distribution in skin layers after application of THC-SLN gel was observed to be significantly higher (*p* ≤ 0.05) as compared with the free THC gel-treated skin. This proves the potential of SLN gel as a suitable drug delivery system to enhance the penetration of THC through the skin.

Table 3 illustrates the numeric values of dermatokinetic parameters obtained from the AUC_0–6h_ (μg · cm^−2^ · h), C_max_ (μg · cm^−2^), T_max_ (h), K_p_, K_e_ and AUC_(0–∞)_ (μg · cm^−2^ · h). The AUC_(0–6h)_ was observed to be significantly higher (*p* ≤ 0.05 each) in the epidermis as compared to the dermis for both systems. However, the AUC_(0–∞)_ was significantly higher (by almost four times) in the epidermis than the dermis for the free drug gel group, showing depleted absorption in the dermis. It is clear from this study that THC-SLN gel permits greater penetration into the deeper layers of skin than the free THC gel. C_max_ was also significantly greater and T_max_ earlier for the THC-SLN gel as compared to free THC gel in both epidermis and dermis. Additionally, it was noted that K_p_ > K_e_ for THC-SLN gel in the epidermis and dermis, thereby allowing more of the drug and longer retention in both skin layers [53,54,55]. The results established that THC-SLN gel has the potential to increase THC skin delivery and enhance its topical bioavailability.

#### 3.4.2. In Vivo Penetration Using Confocal Laser Scanning Microscopy (CLSM)

CLSM is a non-invasive method for observations with high resolution and depth selectivity. Penetration of the hydrophilic fluorescent probe 5,6-carboxyfluorescein (CFC) from the SLN gel was studied in skin layers post treatment with SLNs loaded with CFC, blank SLNs-gel containing CFC or free CFC in Carbopol gel. The particle size and CFC content of dye-loaded nanoparticles were found to be 110.4 nm and 95%, respectively. Treatment with SLN gel loaded with CFC showed penetration of the dye to deeper skin layers, as was evident from the green florescence in the dermis layer at 24 h (Figure 5B image 3) [56]. When free CFC was incorporated to the blank SLN gel, CFC penetration was observed, but more so in the epidermis layer rather than the dermis layer (Figure 5B image 2). Treatment with free CFC in plain gel showed penetration in the upper stratum corneum layer only (Figure 5B image 1). The blank SLN gel hydrated the skin due to its occlusive properties, and thus enhanced penetration of CFC as compared to free CFC in gel, which does not have occlusive properties to enhance skin hydration, and free CFC, being a hydrophilic molecule, shows limited skin penetration. CFC-loaded SLNs in a gel base showed penetration into the deeper layers of skin, and thus would be able to alleviate the inflammation of AD [57,58]. These results are in agreement with previous studies, which have shown that lipid particle-based gels promote deeper penetration and retention of the drug in the skin [59].

### 3.5. Pharmacodynamic Activity

#### 3.5.1. Evaluation of Atopic Dermatitis (AD)

The in vivo anti-inflammatory potential of the THC-SLN gel was assessed in murine models of AD and compared to the commercial product Tacroz^®^ Forte containing 0.1% *w*/*w* tacrolimus, as tacrolimus is a well-established second-line treatment recommended for atopic dermatitis to patients who have not responded to other medication.

AD was induced in mice by applying DNCB topically on the hair-free dorsal side daily for 12 days, as is evident by the SCORAD index (72.6) and the images showing the clinical symptoms of the severity of the disease (Supplementary data Table S1 and Figure S1). The reductions in AD symptoms upon treatment with THC-SLN gel, THC-SLN dispersion, Tacroz^®^ Forte, free THC gel and plain gel were examined with the passage of time (days 0, 13, 15, 18 and 22), and the SCORAD indices were determined (Supplementary data Table S1 and Figure S2). The SCORAD index, developed by the European Task Force on atopic dermatitis, is a well-accepted scoring system for measuring the severity of atopic dermatitis [32,33]. The mice treated with free THC (SCORAD index 24.4 ± 0.58 on 22nd day) showed some alleviation of AD symptoms as compared to positive controls at the end of the treatment period. THC is known for its antioxidant properties, and its scavenging of free radical is one of the important parts of wound healing. It also aids in reducing skin inflammation. However, the skin penetration of free THC is limited and is substantially enhanced when loaded in SLNs. Groups treated with THC-SLN gel and THC-SLN dispersion showed complete mitigation of AD symptoms with SCORAD indices of 0. Reductions in AD were found to be significantly higher (*p* ≤ 0.05) in these groups as compared to the group treated with free THC. This study revealed the bio-enhanced action of THC post incorporation into SLNs to abate AD symptoms. THC-SLNs, being small and having occlusive effects, allow permeation of the drug into deeper layers of skin and retention at the target site for a prolonged period of time, resulting in greater efficacy. The skin healing rapidity and effectiveness of the treatments followed this descending order: THC-SLN gel = THC-SLNs > Tacroz^®^ Forte > blank SLN gel >free THC gel >positive control (Supplementary data Table S2).

It is noteworthy to highlight here that treatment with THC-SLN gel left no prominent scars, nor any indications of erythema, edema, excoriations, crusting/oozing, lichenification or areas affected with dryness. Tacroz^®^ Forte (tacrolimus)-treated groups showed mild edema and papules signifying incomplete healing, having a SCORAD index of 4.7 ± 0.31. Additionally, it is significant to mention here that tacrolimus itself causes itching and burning sensations, which could imply a reason for the partial healing process. On the other hand, THC also exhibits depigmenting properties, along with superior antioxidant and anti-inflammatory effects. The latter is explained by the ability of THC to offer a protective covering on the wound area, by chelating the reactive oxygen species (ROS) and stimulating the wound contraction with its anti-inflammatory effects [60,61]. Further, THC might also promote enhancement in the production of new blood capillaries and fibroblasts. Similar effects have been detailed in another study on wound healing [62]. On the other hand, blank SLN gel (SCORAD index 10.7 ± 0.58 on 22nd day) showed better symptomatic relief than the free THC gel owing to the emollient effect, which promotes healing, as dry skin is one of the main symptoms of AD.

#### 3.5.2. Biochemical Estimation

At the end of treatment (22nd day) biochemical markers, e.g., lipid peroxidation (LPO), glutathione level (GSH) and catalase activity, were estimated [17].

##### Lipid Peroxidation (LPO)

LPO is a distinct marker of ROS linked with oxidation and degradation of polyunsaturated fatty acids in lipid membranes. LPO degradation products include lipid hydroperoxides, conjugated dienes and malondialdehyde (MDA), and have been implicated in the pathology of AD [63].

**Figure 6:**
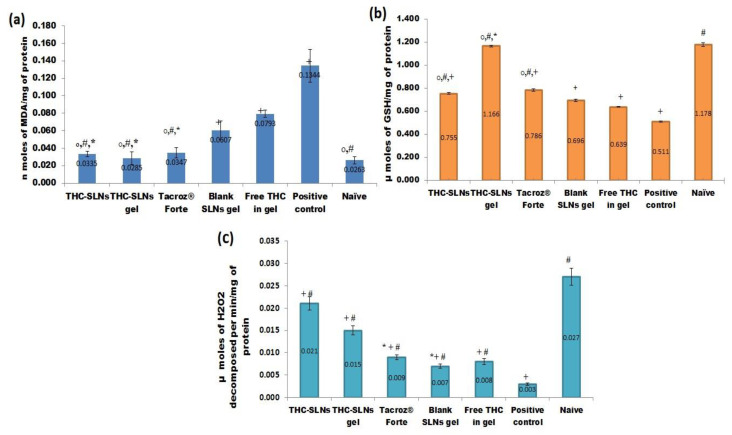
(**a**) Effects of THC-SLN gel, THC-SLN dispersion, Tacroz^®^ Forte, blank SLN gel, free THC gel, positive control and plain gel on lipid peroxidation in the mouse skin of AD models. Values are expressed as mean ± SD (one-way ANOVA followed by Tukey’s test): * denotes insignificant difference at *p* ≤ 0.05 from naïve group; + denotes significant difference as c.f. naïve group; # c.f. positive control; ^o^ c.f. free THC gel group. (**b**) Effects of THC-SLN gel, THC-SLN dispersion, Tacroz^®^ Forte, blank SLN gel, free THC gel, positive control and plain gel on the reduced glutathione levels in the mouse skin of AD models. Values are expressed as mean ± SD (one-way ANOVA followed by Tukey’s test): * denotes insignificant difference at *p* ≤ 0.05 from naïve group; + denotes significant difference as c.f. naïve group; # c.f. positive control; ^o^ c.f. free THC gel group. (**c**) Effects of THC-SLN gel, THC-SLN dispersion, Tacroz^®^ Forte, blank LNs gel, free THC gel, positive control and plain gel on the catalase levels in the mouse skin of AD models. Values are expressed as mean ± SD (one-way ANOVA followed by Tukey’s test): * denotes insignificant difference at *p* ≤ 0.05 from free drug gel group; + denotes significant difference # c.f. positive control; ^o^ c.f. free THC gel group.

The positive control group showed a higher (5.11 times) level of MDA than the naive group. However, treatment groups with THC-SLNs, THC-SLN gel and Tacroz^®^ Forte ointment displayed declines in MDA levels by 75.0, 78.8 and 74.15%, respectively. Furthermore, all these groups showed a complete reversal of elevated MDA levels, and the levels were found to be identical to those of the naïve control group (Figure 6a). Treatment with blank SLN gel, however, reduced the MDA levels by 50.14% only, which is still more than the free THC gel treatment (40.90%) The results clearly reveal the significance of incorporating THC into SLNs.

##### Reduced Glutathione (GSH)

GSH, also called a master antioxidant, is produced by amino acids—for example, glycine, glutamate and cysteine. GSH has the ability to neutralize ROS and provide protection from inflammation. Enhancement of the GSH level neutralizes the ROS and triggers the downregulation of proinflammatory signaling molecules, such as leukotrienes and eicosanoids, to inhibit the pathogenesis of inflammation [64].

Our results revealed a significant reduction in the level of GSH in the positive control group (Figure 6b) as compared to naïve mice (i.e., negative control). THC-SLN dispersion treatment showed an increase in GSH levels by 1.47 times in comparison to the positive control group. On the other hand, THC-SLN gel increased the GSH levels by 2.28 times, in contrast to 1.51 times, on treatment with Tacroz^®^ Forte tacrolimus ointment. The levels of GSH attained with THC-SLN gel were almost twice those of the free THC group, which indicates enhanced skin penetration of THC and better antioxidation by THC post incorporation into SLNs. The increase in GSH levels with blank SLN gel was higher than with free THC gel. This observation is indeed directly related to the antioxidant capabilities of phosphatidylcholine. Previous reports support the effect observed in the study [65].

##### Catalase H_2_O_2_

Catalase is a measure of oxidative stress and depends on the disappearance and breakdown of peroxides. AD patients are more prone to harm caused by ROS, which is noticeable from their higher levels of MDA and lower levels of enzymatic and non-enzymatic antioxidants, such as catalase [66]. It was observed that catalase levels were lower in the positive control group by nine times as compared to naive mice (Figure 6c). With THC-SLN gel treatment, catalase levels were 0.56 times lower than in the naïve skin, five times higher than in the positive control and 1.67 times higher than in the group treated with free drug gel. Catalase levels were 1.9 times greater for THC-SLN gel treatment in comparison with treatment with the tacrolimus ointment Tacroz^®^ Forte. These results are in agreement with previous results establishing the superior efficacy of THC when loaded onto lipid nanoparticles compared to being given as a free drug; and this treatment showed better recovery than the current treatment with tacrolimus ointment.

#### 3.5.3. Molecular Markers (Interleukins (IL-6) and TNF-α) in Skin Homogenates

The changes in levels of IL-6 and TNF-α were assessed in the skin homogenates obtained on the 22nd day at the end of treatment for all groups.

##### Effect on the Level of TNF-α

**Figure 7:**
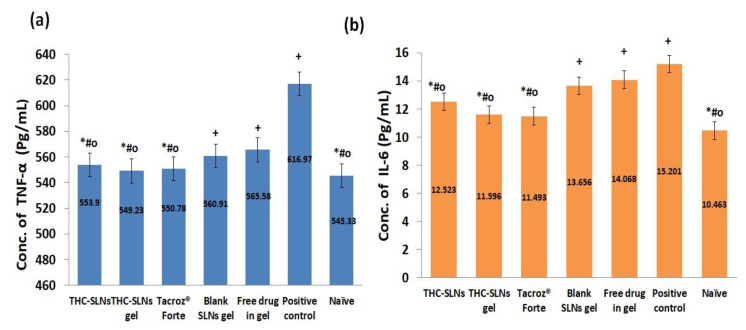
(**a**) Comparative bar graph of concentrations (pg/mL) of TNF-α for the different groups. Values are expressed as mean ± SD (one-way ANOVA followed by Tukey’s test): * denotes insignificant difference at *p* ≤ 0.05 from naïve group; + denotes significant difference as c.f. naïve group; # c.f. positive control at *p* ≤ 0.05; ^o^ c.f. free THC gel group at *p* ≤ 0.01. (**b**) Comparative bar graph of concentrations (pg/mL) of IL-6 for the different groups. Values are expressed as mean ± SD (one-way ANOVA followed by Tukey’s test): * denotes insignificant difference at *p* ≤ 0.05 from naïve group; + denotes significant difference as c.f. naïve group; # c.f. positive control; ^o^ c.f. free THC gel group at *p* ≤ 0.05.

The level of TNF-α was found to be 616.97 ± 8.84 pg/mL in the positive control group, significantly higher (*p* ≤ 0.05) compared to the THC-SLN gel group (549.23 ± 6.74 pg/mL) and the naïve group (545.33 ± 15.55 pg/mL) by 1.12 and 1.13 times, respectively (Figure 7a). The groups treated with THC-SLN dispersion (553.90 ± 1.35 pg/mL) and Tacroz^®^ Forte ointment (550.78 ± 4.05pg/mL) did not show any significant differences in level of TNF-α as compared to the naive control group. Free THC gel (565.58 ± 3.57 pg/mL) showed higher levels of TNF-α than THC-SLN gel (549.23 ± 6.74 pg/mL) and blank SLN gel groups (560.91 ± 3.57 pg/mL). The latter may be attributed to the antioxidant effects of the phosphatidylcholine present in the SLNs. These results are a proof of the improved efficacy of THC when delivered via SLNs to inflamed skin, even at the molecular level. Further, achieving similar results in alleviating TNF-α both with Tacroz^®^ Forte and with THC-SLN gel is the highlight of the current investigation. This gives clear evidence for the development of a safer product with equivalent efficacy to the marketed product, Tacroz^®^ Forte ointment, which is associated with serious side effects with prolonged use [67].

##### Effect on the Level of IL-6

IL-6 is associated with cell and membrane destruction at the site of injury, and thus is identified as a chemical signal for the infiltration of other anti-inflammatory cells [68]. The levels of IL-6 were found to be 15.20 ± 0.78 pg/mL in the positive control group, which were significantly higher (*p* ≤ 0.05) than those of the THC-SLN gel group (11.59 ± 0.82 pg/mL) and naïve group (10.46 ± 0.99 pg/mL) by 1.31 and 1.45 times, respectively. The groups treated with THC-SLN dispersion (12.53 ± 0.82 pg/mL) and Tacroz^®^ Forte (11.49 ± 0.47 pg/mL) showed decreases in IL-6 from the positive control by 1.21 and 1.32 times, respectively (Figure 7b)**.** However, Tacroz^®^ Forte showed a similar decrease in the level of IL-6 to that achieved post treatment with THC-SLN gel. Free THC gel treatment (14.07 ± 0.93 pg/mL) showed IL-6 levels 1.21 times greater (*p* ≤ 0.05) than after THC-SLN gel treatment, and there was an insignificant difference (*p* ≤ 0.05) between IL-6 in the former group and IL-6 after treatment with blank SLN gel (13.67 ± 0.89 pg/mL).

High levels of pro-inflammatory cytokines, i.e., TNF-α and IL-6, elevate the infiltration of macrophages and basophils into the dermis layer [69]. As expected, marketed ointment and SLN-based formulations showed reductions in levels of TNF-α (*p* ≤ 0.01, one-way ANOVA) and IL-6 (*p* ≤ 0.05, one-way ANOVA) as compared to the free drug gel. It was concluded that it is a potential candidate to prevent the pathological increases in the levels of various pro-inflammatory cytokines in AD disease [70].

#### 3.5.4. Histopathological Investigation

**Figure 8:**
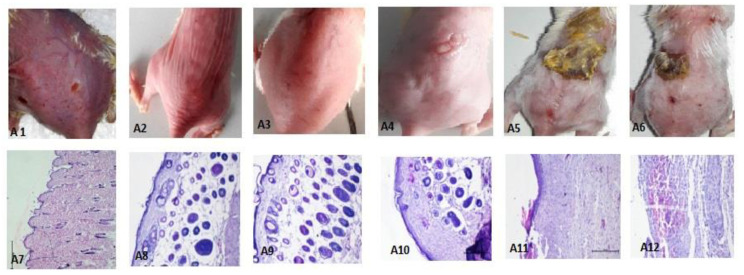
The photographs revealing the appearance of mouse skin (**upper** panel) and histopathological sections of mouse skin (**lower** panel) from various treatment groups (murine DNCB-induced atopic dermatitis models): positive control (**A1**,**A7**), THC-SLN dispersion (**A2**,**A8**), THC-SLN gel (**A3**,**A9**), Tacroz^®^ Forte ointment (**A4**,**A10**), free THC gel (**A5**,**A11**) and blank SLN gel (**A6**,**A12**).

Histopathological observations of skin treated with DNCB only (positive control) showed full-thickness necrosis and inflammatory cell infiltration, along with pus exudates. Dark clusters of microorganisms were also spotted (Figure 8(A7)). In some animals, the edge of an ulcer also showed the degeneration of basal cells in the epidermis. Histopathological assessments of the groups treated with THC-SLN dispersion and gel are depicted in Figure 8(A8,A9), revealing full recovery with skin histology equivalent to that of the normal group. Epidermis and dermis layers with dermal appendages, sebaceous glands and hair follicles in the fat and deeper layer of muscles were found to be normal. Longitudinal divisions of hair follicles were present as in normal skin. The Tacroz^®^ Forte tacrolimus ointment treatment group showed good recovery in most parts of the skin, but some areas showed inflammatory cells in the epidermis, signifying partial recovery (Figure 8(A10)).

In the free THC gel treatment group, ulceration was still observed on the skin. The squamous epidermis was found to be normal in some parts but missing in other parts of the skin, signifying partial recovery. The dermis layer showed inflammation penetrating to the muscular layer and the absence of the dermal appendages. Inflammatory cells with plump spindle cell fibroblasts were also observed, signifying early healing, as shown in Figure 8(A11). Another area showed inflammatory cells, red cells and a nerve trunk. Finally, another area illustrated full-thickness necrosis with damage to all skin layers and dense inflammatory cells.

The blank SLN gel treatment group showed healing to some extent, but ulcers were observed along with necrotic eschar (Figure 8(A6,A12)). Inflammatory cells emphasizing chronic inflammation, including lymphocytes, monocytes, macrophages and a chain of multinuclear giant cells of the “foreign body” types, were observed. A dense layer of acute inflammation and hemorrhage with edema in some capillaries and fibroblasts was also detected. Regeneration of epidermis showed knots of darkly stained basal cells.

These histopathological studies confirmed the effectiveness of developed THC-SLN gel for the treatment of inflammation in AD.

## 4. Conclusions

In this research, we have successfully developed THC-SLN gel using a microemulsification technique with high drug entrapment to improve THC’s penetration into the deeper layers of the skin for treatment of AD. NTA confirmed the polydisperse nature of the particles, which had a modal particle size of 109.2 nm based on the Brownian movement of individual particles. FTIR confirmed that THC had been successfully loaded into the SLN dispersion. THC-SLN gel revealed occlusive properties with skin hydration potential equivalent to that of marketed ointment and 1.44 times that of free THC. Dermatokinetic studies demonstrated enhanced topical bioavailability in both epidermis and dermis with THC-SLN gel as compared to free THC gel. CLSM clearly illustrated deeper skin penetration of the SLN-loaded gel, with maximum fluorescence intensity in the dermis layer, whereas fluorescence from the free drug gel concentrated mostly in the top stratum corneum layer. THC displayed enhanced bioactivity post incorporation into SLNs, when studied in a murine model of AD. Histopathological studies further established complete healing of skin on application of THC-SLN gel and alleviation of the symptoms of AD. Additionally, the biochemical evaluation established its antioxidant potential, as there was a statistically significant reduction (*p* ≤ 0.05) in the MDA level (4.71 times), alongside enhancements in GSH (2.28 times) and catalase (5 times) levels vis-a-vis positive control. The alleviation was significantly different (*p* ≤ 0.05) from that produced by Tacroz^®^ Forte or free THC gel. The THC-SLN gel revealed significant reductions in the levels of cytokines IL-6 and TNF-α, exhibiting its anti-inflammatory efficacy. Thus, THC-SLNs are a promising future treatment for AD with the likely ability to overcome the limitations of current therapies. These results encourage further investigation for exploiting the use of this therapeutic tool and should nurture further clinical investigation in this specific inflammatory skin disease.

## Figures and Tables

**Table 1:** Various groups along with their treatments for the pharmacodynamic study in murine a atopic dermatitis model.

Groups	Formulation
1	THC-SLN dispersion
2	THC-SLN gel
3	Tacroz^®^ Forte (0.1% *w*/*w* tacrolimus ointment)
4	Blank SLN gel
5	Free drug gel
6	Positive control (DNCB, 0.5% *w/v* in a 3:1 of acetone: olive oil)
7	Negative control (Naive)

**Table 2:** Comparative skin hydrating potential after various treatments in *Lacca* mice.

S.No.	Formulation	Thickness of Skin (cm) (*n* = 3)	Skin Hydration Potential (SHP)
1	THC- SLN gel	0.13 ± 0.01	216.7 ± 16.70
2	THC-SLN dispersion	0.12 ± 0.01	200 ± 16.70
3	Free THC gel	0.09 ± 0.02	150 ± 33.30
4	Blank SLN gel	0.10 ± 0.02	166.7 ± 33.30
5	Tacroz^®^ Forte,	0.12 ± 0.01	200 ± 16.70
6	Control skin (naïve)	0.06 ± 0.01	100 ± 16.70

All values expressed as mean ± SD (*n* = 3).

**Table 3:** Various dermatokinetic parameters from concentration time profiles in the epidermises and dermises of Wistar rats after treatment with THC-SLN gel and free THC gel.

S.No.	Dermatokinetic Parameters	THC-SLNs Gel		Free THC Gel	
		Epidermis	Dermis	Epidermis	Dermis
1.	AUC_0–6h_ (μg cm^−2^ h)	149.56 ± 0.028	136.21 ± 0.071	68.75 ± 0.097	23.35 ± 0.072
2.	AUC_0–∞_ (μg cm^−2^ h)	157.09 ± 0.059	141.94 ± 0.069	147.63 ± 0.073	41.40 ± 0.071
3.	C_max_ (μg cm^−2^)	52.98 ± 0.035	57.51 ± 0.036	33 ± 0.045	20.01 ± 0.040
4.	T_max_ (h)	0.56 ± 0.026	0.48 ± 0.020	1.09 ± 0.025	1.02 ± 0.027
5.	K_p_	6.72 ± 0.049	4.14 ± 0.043	0.66 ± 0.050	0.44 ± 0.055
6.	K_e_	0.16 ± 0.087	0.33 ± 0.089	3.75 ± 0.094	4.48 ± 0.076

All values expressed as mean ± SD (*n* = 3).

## Data Availability

The data presented in this study are available in this article.

## Supplementary Materials

The following supporting information can be downloaded at: https://www.mdpi.com/article/10.3390/nano12040636/s1. Figure S1: Representative clinical atopic dermatitis symptoms on various days. Table S1: SCORAD for atopic dermatitis induced via DCNB in mouse models, wherein therapeutic intervention started on day 13 with THC-SLN gel, THC-SLNs, Tacroz^®^ Forte, free THC gel or blank SLN gel. Figure S2: Representative images of the successive treatment from day 13 to day 22 post treatment with (A) THC-SLNs, (B) THC-SLN gel, (C) Tacroz^®^ Forte, (D) free THC gel or (E) blank SLN gel. Table S2: Percentages of healing for all groups on different days of observation (*n* = 4).
