# Supplementary material for: Tetrahydrocurcumin Lipid Nanoparticle Based Gel Promotes Penetration into Deeper Skin Layers and Alleviates Atopic Dermatitis in 2,4-Dinitrochlorobenzene (DNCB) Mouse Model
Source: Nanomaterials (Basel). 2022 Feb 14;12(4):636. doi: 10.3390/nano12040636 (PMC8879523; doi:10.3390/nano12040636)
Supplement: Supplementary file 1 [file nanomaterials-12-00636-s001.zip › nanomaterials-1367686-supplementary.pdf]

nanomaterials

Supplementary Materials

Tetrahydrocurcumin Lipid Nanoparticle based Gel Promotes Penetration into Deeper Skin Layers and Alleviates Atopic Dermatitis in 2,4-Dinitrochlorobenzene (DNCB) Mouse Model

Komal Saini [1,2], Nancy Modgill [1], Kamalinder K. Singh [2,3,4,*] and Vandita Kakkar [1,*]

1 Department of Pharmaceutics, University Institute of Pharmaceutical Sciences, Panjab University, Chandigarh 160014, India; komalsainiks@gmail.com (K.S.); nancymodgill92@gmail.com (N.M.)
2 School of Pharmacy and Biomedical Sciences, Faculty of Clinical and Biomedical Sciences, University of Central Lancashire, Preston, Lancashire PR1 2HE, UK
3 UCLan Research Centre for Smart Materials, University of Central Lancashire, Preston, Lancashire PR1 2HE, UK
4 UCLan Research Centre for Translational Biosciences and Behavior, University of Central Lancashire, Preston, Lancashire PR1 2HE, UK
* Correspondence: ksingh1@uclan.ac.uk (K.K.S.); vanditakakkar@yahoo.co.in (V.K.)

SCORAD (Scoring Atopic Dermatitis)

Effectiveness against atopic dermatitis was assessed in terms of SCORAD points. It is the most commonly used scoring system developed by the European Task Force on atopic dermatitis in 1993 for measuring the severity of atopic dermatitis. It is used to standardize the assessment of atopic dermatitis and to help in the interpretation of therapeutic effects of the treatment under investigation.

The SCORAD index is a composite score based on 3 sub-scores:

A = The extent score based on body surface area calculated using the 'Rule of 9',

B = Intensity score based on 6 clinical findings in atopic dermatitis (namely erythema, edema or papulations, oozing or crusting, excoriation, lichenification, dryness) graded on a scale of 0-3 (0- absent, 1- mild, 2- moderate, 3- severe),

C = The score for pruritus and sleep loss graded on a visual analogue scale of 0 to 10. The severity is based on the average extent for the last 3 days. The formula for SCORAD calculation is given below:

SCORAD = $A/5 + 7(B/2) + C$

SCORAD values were evaluated for the groups in which atopic dermatitis was induced starting from the day 1 and their subsequent treatment with the groups shown in figure and table, beginning from the day 13 as given below. Based on the SCORAD values, percentage healing was assessed for all the groups up to day 22nd of the protocol.

Table S1. SCORAD for atopic dermatitis induced via DCNB in mice model, wherein therapeutic intervention starts on day 13th with THC-SLNs gel, THC-SLNs, Tacroz® Forte, free THC gel and blank SLNs gel respectively.

Days	SCORAD					
	Positive control	THC-SLNs gel	THC-SLNs	TACROZ® FORTE	Free THC gel	Blank SLNs gel
1	10.2 ± 1.00	10.2 ± 0.58	10.2 ± 1.25	10.2 ± 0.58	10.2 ± 1.15	10.2 ± 0.58
2	13.7 ± 0.58	13.7 ± 0.58	13.7 ± 0.58	13.7 ± 1.16	13.7 ± 0.58	13.7 ± 0.58
3	17.4 ± 0.65	17.4 ± 0.37	17.4 ± 0.83	17.4 ± 1.37	17.4 ± 0.86	17.4 ± 1.53
4	37.9 ± 1.01	37.9 ± 1.15	37.9 ± 0.58	37.9 ± 1.24	37.9 ± 1.16	37.9 ± 1.53
5	41.6 ± 0.87	41.6 ± 0.86	41.6 ± 0.76	41.6 ± 1.67	41.6 ± 0.38	41.6 ± 0.85
6	34.6 ± 0.59	34.6 ± 1.23	34.6 ± 1.12	34.6 ± 0.89	34.6 ± 0.49	34.6 ± 0.46
8	48.1 ± 1.53	48.1 ± 1.15	48.1 ± 0.58	48.1 ± 1.16	48.1 ± 1.16	48.1 ± 0.58
9	62.1 ± 1.53	62.1 ± 1.15	62.1 ± 0.58	62.1 ± 0.58	62.1 ± 1.16	62.1 ± 0.58
10	69.1 ± 0.41	69.1 ± 1.35	69.1 ± 1.71	69.1 ± 0.67	69.1 ± 0.58	69.1 ± 0.95
12	72.6 ± 1.16	72.6 ± 2.01	72.6 ± 0.58	72.6 ± 0.58	72.6 ± 1.16	72.6 ± 1.16
13	69.6 ± 1.73	45.1 ± 0.58	48.6 ± 1.21	45.1 ± 1.16	52.1 ± 0.58	44.9 ± 2.31
14	66.1 ± 1.16	34.4 ± 1.15	41.4 ± 0.58	41.4 ± 0.58	45.1 ± 1.16	34.4 ± 1.16
15	52.1 ± 1.27	20.9 ± 0.87	27.9 ± 0.58	24.4 ± 0.74	34.9 ± 1.32	24.4 ± 1.25
18	48.6 ± 1.16	17.2 ± 0.58	17.2 ± 0.62	20.7 ± 0.58	27.9 ± 1.73	20.7 ± 1.53
20	43.7 ± 1.16	7.2 ± 0.59	10.7 ± 0.63	10.2 ± 0.58	27.9 ± 1.53	14.2 ± 0.58
22	40.2 ± 0.58	0	0	4.7 ± 0.31	24.4 ± 0.58	10.7 ± 0.58

Table S2. Percentage healing of all groups on different days of observation (n = 4).

Days	THC- SLNs gel	THC- SLNs	TACROZ® FORTE	Free THC gel	Blank SLNs gel	Positive control
12	0	0	0	0	0	0
13	37.9 ± 0.97	33.1 ± 1.16	37.9 ± 0.97	28.23 ± 3.89	38.2 ± 1.95	4.1 ± 2.14
14	52.6 ± 1.94	42.9 ± 1.95	42.9 ± 2.92	37.9 ± 2.89	52.6 ± 2.92	8.95 ± 1.17
18	76.3 ± 0.78	76.3 ± 0.97	71.5 ± 3.89	61.6 ± 0.97	71.5 ± 0.97	33.1 ± 1.75
20	90.1 ± 0.97	85.3 ± 0.98	86.0 ± 0.98	61.6 ± 1.95	80.4 ± 3.89	39.8 ± 0.97
22	100.00	100.00	93.5 ± 0.97	66.4 ± 2.92	85.3 ± 1.94	44.6 ± 0.97

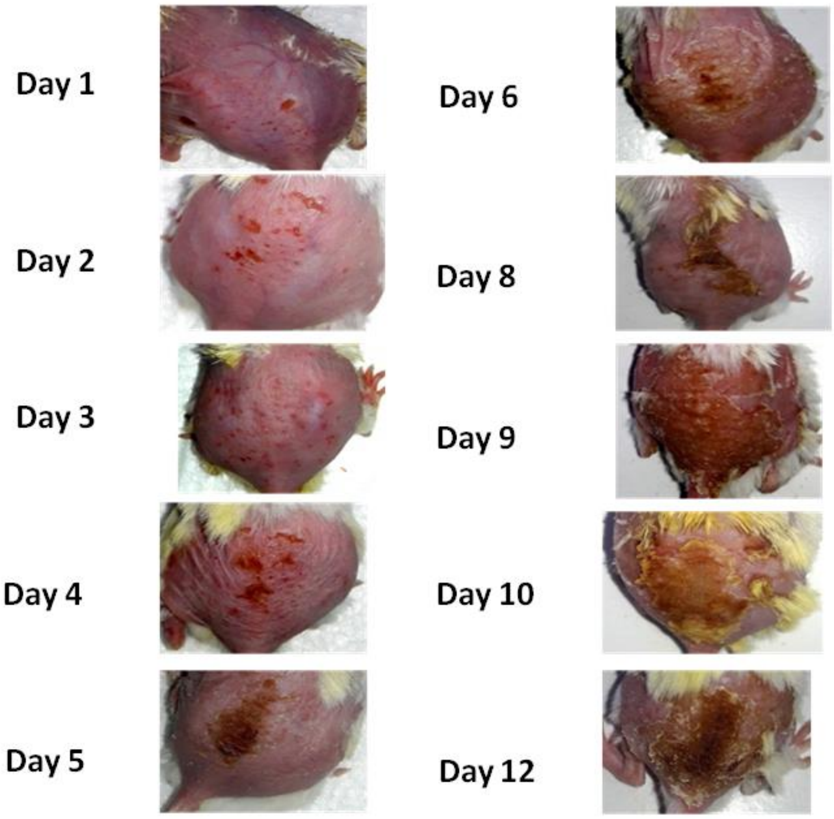

Figure S1. Representative clinical atopic dermatitis symptoms on various days.

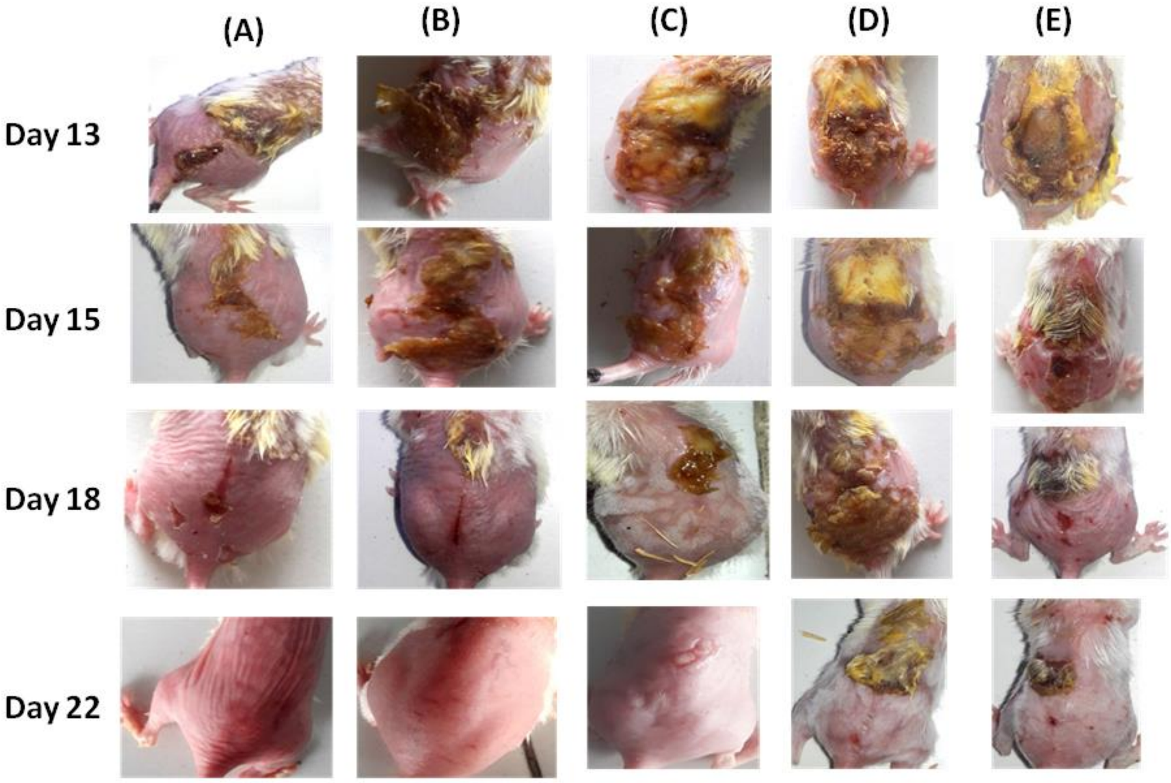

Figure S2. Representative images of the successive treatment from day 13th to day 22nd post treatment with (A) THC-SLNs (B) THC-SLNs gel (C) Tacroz® Forte (D) Free THC gel and (E) blank SLNs gel respectively.
